# Retention Force of Removable Partial Dentures with CAD-CAM-Fabricated Telescopic Crowns

**DOI:** 10.3390/ma13143228

**Published:** 2020-07-20

**Authors:** Christin Arnold, Ramona Schweyen, Arne Boeckler, Jeremias Hey

**Affiliations:** Department of Prosthodontics, Martin-Luther-University Halle-Wittenberg, Magdeburger Str. 16, 06112 Halle, Germany; christin.arnold@uk-halle.de (C.A.); arne.boeckler@zahnzentrum-halle.de (A.B.); jeremias.hey@uk-halle.de (J.H.)

**Keywords:** removable partial denture, retention force, double crowns, friction, wear, CAD-CAM

## Abstract

The aim of this study was to compare the retention forces after artificial aging of telescopic crowns fabricated either by a conventional lost-wax technique or by computer-aided design/computer-aided manufacturing (CAD-CAM) technology. Two types of telescopic crowns (0°) were fabricated conventionally using high-noble (group A) and non-precious metal (group B). CAD-CAM fabricated telescopic crowns (0°) were made of non-precious metal (group C). Retention forces were assessed before and after artificial aging and after axial and non-axial loading. Initially [I.] and after artificial aging [IV.], specimens of group C (I. 16.2 N; IV. 13.6 N) exhibited the highest retention forces. The retention forces in groups B (I. 12.5 N; IV. 4.6 N) and A (I. 9.6 N; IV. 2.9 N) were found to be lower than those of group C. The retention force differences between the groups were statistically significant (*p* < 0.001) at all measurement times [I. vs. IV.]. Different fabricated telescopic crowns with approximately identical designs and materials exhibited different retention forces and different long-term retentive behavior. An optimized CAD-CAM process with individually defined design parameters ensured telescopic crowns with a better fit. These findings might influence prospective clinical decision-making.

## 1. Introduction

The combination of removable and fixed dentures with double crowns as anchoring elements, both on natural abutments and implants, has proven itself over decades as a treatment modality [1,2,3,4,5]. Their advantages include circular and rigid support of the abutment teeth, adequate retention, their supporting and leading function combined with acceptable aesthetics, simple handling and cost-effective extensibility [3,4]. A common type of double crown is the telescopic crown. A telescopic crown, in its original version, has parallel sided walls with a flank angle very close to 0°. Retention is achieved by the large contact surfaces between the primary and secondary crowns. In the original version, high-gold alloys were used for the primary and secondary crowns. Due to the comparatively low modulus of elasticity of these alloys, they allow a simple conventional production by means of casting technology and a comparatively uncomplicated adjustment of the desired holding force chairside. In recent decades, gold prices have risen sharply. For this reason, alternative materials were used and many designs have been developed for the concept of double crowns. Along with variations in design, the various material compositions influence the retentive behavior of double crowns [6]. For the production of classic, parallel-milled (0°) telescopic crowns, CoCrMo alloys can be considered an alternative to the high-gold alloys [6]. The conventional processing (lost-wax technique) of these alloys is susceptible to deficiencies due to high casting temperatures and easy oxidation. The high modulus of elasticity renders manual processing and the adjustment of the retention force more difficult. Optimal retention behavior between primary and secondary crowns is only ensured by a transition fit with minimum clearance. The error tolerance for the undesired press or clearance fit is in the micrometer range. The fabrication of well-functioning telescope prostheses from CoCrMo alloys requires a high level of knowledge and practical skills. 

Digital technologies are characterized by high precision, even in the micrometer range, when parameters are selected correctly [7]. Furthermore, an increase in efficiency is attributed to the use of CAD-CAM processes, which avoids errors associated with casting technology [8,9]. Therefore, they are successfully used for the fabrication of many types of dentures. However, the fully computer-assisted fabrication of double crowns-both primary and secondary crowns—is currently not widespread. The precise adjustment of the fitting tolerance proves to be problematic. The translation of the dental technician’s practical experience into algorithms is a challenge. New milling strategies should now have made improvement possible and should bring about a retention behavior like that achieved by conventional fabrication.

The aim of this study was to compare the retention forces of telescopic crowns exclusively fabricated by using CAD-CAM and those of analogously fabricated, structurally identical telescopic crowns (original type, 0°), before and after artificial aging. As a null hypothesis, it was assumed that the different fabrication processes and materials, respectively, do not have any effect on the retention behavior of the telescopic crowns.

## 2. Materials and Methods 

**Figure 1:**
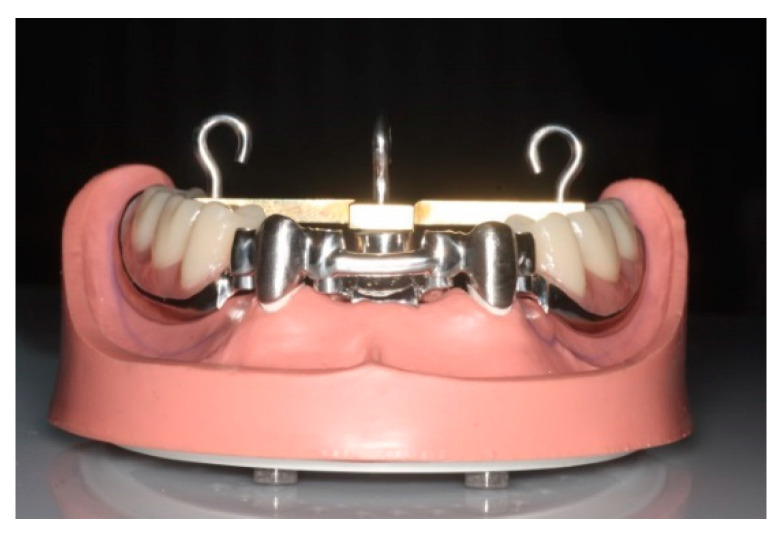
Test model including test prosthesis for conventionally fabricated pairs of double crowns and T-shaped traverse with hook for anchoring to the universal testing machine.

The test model was developed in a previous investigation and based on a study model of the lower jaw with teeth 33 and 43 (T2, KaVo Dental, Biberach, Germany) remaining (Figure 1) [6]. Preparation of the canines was performed in accordance with the requirements for double crowns (6°/3°). The model was modified so that duplicated stumps made of epoxy resin (epoxy resin; Tri-Dynamics Tri-Epoxy, Keystone Industries, Cherry Hill, UT, USA) and equipped with threaded rods on the bottom side, could be reversibly fixed in either a rigid or elastic way. In order to realize this, two hollow cylinders made of stainless steel were fixed firmly in corresponding holes in the model base [6]. Duplicated stumps were fixed within the model base with two nuts. To prevent rotation, both hollow cylinders provided recesses for the uptake of corresponding notches located on the distal side of the duplicated stumps. Two different placeholders could be inserted under the duplicated stumps to simulate rigid or elastic fixation—a rubber O-Ring for elastic fixation and a customized high-strength plastic cylinder for rigid fixation. Elastic fixation allowed an axial movement of the stumps of 200 µm at a load of 5 N.

A rubber gingival mask was adapted on the model base to simulate mucosal tissue. The completed phantom model was placed into a salivary bath that could be fixed axially in all testing machines (artificial saliva [Glandosane, Cell Pharm, Hannover, Germany] and distilled water [ratio 2:1]) [6].

A universal testing prosthesis fitting the modified phantom model was fabricated for the insertion of the secondary crowns. In the test prosthesis, the free-end saddles were completely set in metal. On the free-end saddles, the second premolar as well as the first and second molars were replaced by prefabricated plastic teeth. In the middle, at a distance of 15 mm from each of the secondary crowns, a support with an internal thread was created for fastening a T-shaped traverse. At a distance of 26 mm from each of the secondary crowns, two further supports with an internal thread were incorporated into the occlusal surfaces of teeth 36 and 46 to fasten a T-shaped traverse. The T-shaped traverse was fixed onto the test prosthesis to determine points of defined mechanical strain and removal (Figure 1) [6]. This was ensured by depressions with a diameter of 3 mm to accommodate a punch of the loading machine or by fixing hooks in the center of the depression.

### 2.1. Groups A and B

The telescopic crowns of groups A and B were fabricated conventionally by an experienced dental technician of a certified dental laboratory (Rübeling Dental-Labor, Bremerhaven, Germany) using the lost-wax technique. For each type of double crown, five specimens (prostheses) were fabricated.

#### 2.1.1. Primary Crowns

The primary crowns were modelled in wax directly on the duplicated (prepared) stumps on the KaVo-model (S–U–Dipping wax yellow, Schuler–Dental, Ulm, Germany). The embedding of the wax patterns was carried out alloy-specifically (group A: Heravest Saphir, Kulzer GmbH, Hanau, Germany, D; group B: EHT- Superfine, SRL Dental GmbH, Ludwigshafen, Germany, D). Subsequently, the casting muffle was preheated and the wax burned out (Mihm-Vogt Laborofen, MIHM-Vogt GmbH Co. KG, Stutensee-Blankenloch, Germany, D). After casting, the precious crowns were blasted at 2 bar and the non-precious crowns at 4 bar. The parallel side walls were finished with a 0° cutter (H364RE.023, 10,000 RPM; H364RF.023, 3000 RPM, Komet Dental, Lemgo, Germany) and polished (goat-hair brush, diamond paste, SAE Dental, Bremerhaven, Germany) [6, groups A and B]. Since the size of the contact surfaces between primary and secondary crown is decisive, the parallel surfaces of all primary crowns were limited to 7 mm height labially, 5 mm approximally and 4 mm lingually, corresponding to an anatoform design. Vestibular the primary crowns had a width of ~4 mm and proximal of ~6 mm. A thickness of the primary crowns of ~0.5 mm was selected according to clinical practice. The thickness was checked manually with a caliper.

Primary crowns were fixed on the prepared stumps using composite cement (Nimetic Cem, 3M ESPE, Seefeld, Germany). To avoid excessive retention due to incorrect cementation it was deviated from the clinical procedure. In this study, all primary crowns were cemented onto the stumps after fabrication. The cementation was performed manually by the dental technician in a predefined and standardized way. After cementation of the primary crowns, the secondary crowns were fabricated.

#### 2.1.2. Secondary Crowns

The secondary crowns were modelled in resin directly on the rigidly fixed and cemented primary crowns (Pattern Resin, LS, GC, Alsip, IL, USA). Since—depending on the modulus of elasticity—the alloy has an influence on the retention, the thickness of the secondary crowns was determined to ~0.6 mm. To simulate splinting, the two secondary crowns were connected by a bar. Four screw connections were installed proximally to the crowns on the bar to fix it with a universal testing prosthesis (Figure 1). As described in Section 2.1.1., investment material was chosen in accordance with the alloy composition (Table 1). After casting and blasting (2 or 4 bar), the secondary crowns were fitted manually by the dental technician using a milling machine and rubber polisher (H77NEX.104.023 VPE 5, 9702F.900.060 VPE 10 Gebr. Brasseler GmbH&Co.KG, Lemgo, Germany, D) inner side of the secondary crowns.

### 2.2. Group C 

#### 2.2.1. Primary Crowns

The primary crowns of group C were fabricated by a certified dental laboratory (Dentallabor Rübeling und Klar GmbH Berlin, Germany). Duplicated stumps were scanned (Desktopscanner D900 3Shape A/S, Copenhagen, Denmark). The primary crowns were designed virtually (Dental Designer (3Shape Dental System 2015—Premium, 3Shape A/S, Copenhagen, Denmark) and milled by a 5-axis milling machine (Organical 5XT R+K CAD/CAM Technologie GmbH Co. KG, Berlin Germany). Following the conventional approach, the dimensions of the parallel surfaces of the primary crowns were transferred to the virtual construction. After fitting on the parallel sided walls, the telescopic crowns were finished with a 0° cutter (H364RE.023, 10,000 RPM; H364RF.023, 3000 RPM, Komet Dental, Lemgo, Germany). All primary crowns were polished (goat-hair brush, diamond paste, SAE Dental, Bremerhaven, Germany). The primary crowns were fixed on the prepared stumps using composite cement (Nimetic Cem, 3M ESPE, Seefeld, Germany).

#### 2.2.2. Secondary Crowns

The secondary crowns of group C were fabricated by the CAD-CAM production center at Mack Dentaltechnik GmbH (Dornstadt, Germany). The stumps and the primary crowns were scanned optically (Desktopscanner Iscan D104i max. resolution 0.005 mm, Imetric 4D Imaging Sàrl, Courgenay, Switzerland) as well as by tactile scanning (contact scanner DS10, Renishaw plc, Gloucestershire, UK). The scan data were then compared. Analogous to conventional fabrication, the thickness was ~0.6 mm labially and lingually. This was limited by design parameters. The secondary crowns were constructed and milled in one unit with a simplified testing prosthesis (Exocad GmbH, Darmstadt, Germany; version 5164). On the bottom of the prosthesis, three contact areas at 36, 46 and centrally between 31 and 41 were created. On the upper side of the prosthesis, hollow cylinders were designed in identical positions to accommodate the punch of the loading machines and the internal threads for the T-shaped traverse. The distance between the tip of the telescopic crowns and the center of the hollow cylinders was also 26 mm and 15 mm, respectively. 

The fit parameters (internal geometry design) for the friction surface between the primary and secondary crowns were optimized by the company. As with conventional production, the aim was, based on the clinical experience of the company or the dental technician, to create the best possible retention forces. They are generated using individually programmed milling paths (CAM software hyperDENT V7.0 [FOLLOW-ME Technology GmbH, Munich, Germany]). The 5-axis milling machine DMG Ultrasonic 10 (DMG MORI, Bielefeld, Germany), with Mack high performance solid carbide milling (torus, ball and grinding pins) for dental applications, was used to mill the prosthesis structure, including the secondary crowns, in two stages. After the first stage, the secondary crowns remained in the blank. The fit was then checked. During this process, the primary crowns must slide smoothly into the secondary crowns and, after a final joining pressure, adhere to the respective secondary crown by sufficient friction. In the second stage, the internal geometry was re-milled by 5 µm. Finally, the prostheses with the secondary crowns were separated from the blank.

For each type of telescopic crown (Table 1), five specimens (n = 15 prostheses with 30 telescopic crowns) were fabricated.

### 2.3. Test Procedure

All experiments were performed in a salivary bath (Glandosane, Cell Pharm, Hannover, Germany and distilled water in a ratio of 2:1) at constant ambient temperature (22 ± 2 °C). The load and draw-off directions were strictly axial, perpendicular to the model base and parallel to the insertion direction.

### 2.4. Retention Force Measurement

Analogous to Arnold et al. [6], the retention force measurements were performed using a universal testing machine (Z010, Zwick Roell, Ulm, Germany). The stumps were fixed rigidly. Before removal, the testing prosthesis was applied onto the test model with a pressure force of 50 N. For each pair of telescopic crowns, 20 removals were performed at each measuring point (preload 0.1 N; velocity 50 mm/min; 3 mm change in height). Mean values were used.

### 2.5. Artificial Aging

The simulation of non-axial wearing movements was performed in a loading machine. The stumps were fixed elastically. Non-axial loading was performed in the area of the first molars using a hemispherical punch. In each quadrant, a total of 25,000 cycles were performed (1 Hz velocity and 50 N masticatory force).

The simulation of axial wearing movements was performed in the aforementioned universal testing machine. The stumps were fixed rigidly. A rigid connection between the center of the universal testing prosthesis’ T-shaped traverse and a punch fixed on the upper traverse of the universal testing machine was established. For each specimen, 15,000 insertion and removal cycles were performed (10 mm/s velocity, 3 mm change in height, 20 N axial load).

**Figure 2:**
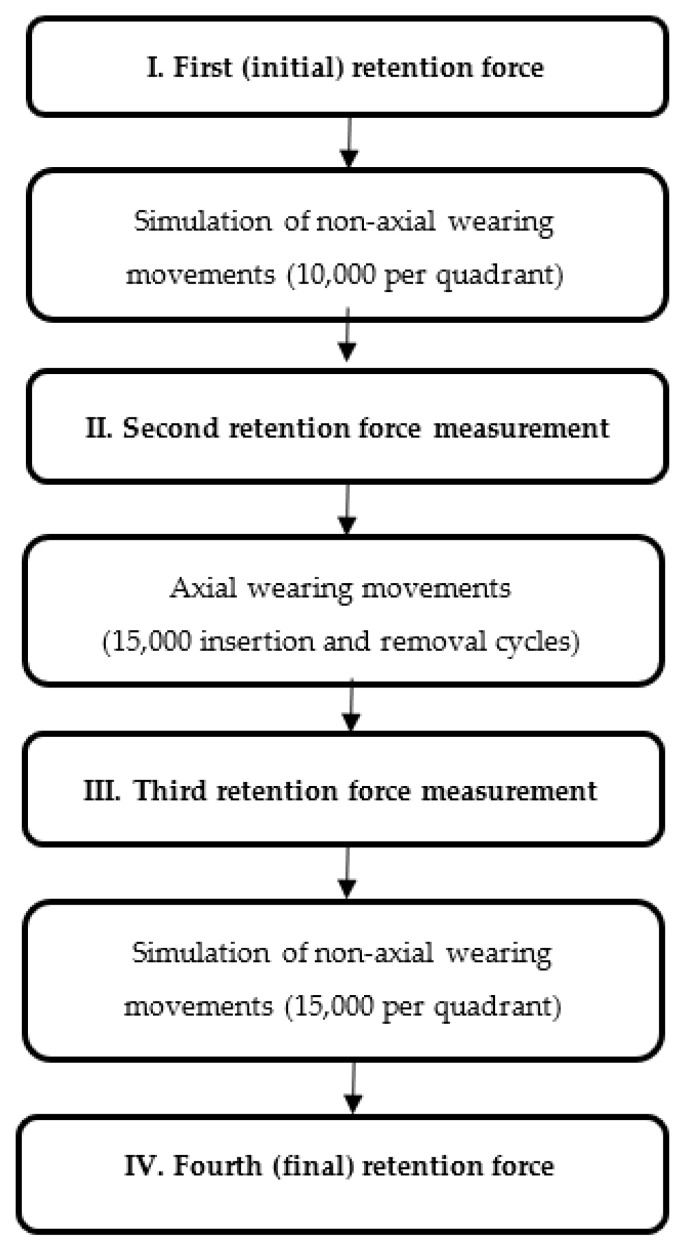
Test procedure. After initial retention force measurement, non-axial wearing movements were simulated (10,000 cycles per quadrant) followed by second retention force measurement. Afterwards, axial loading was simulated in a universal testing machine (15,000 cycles) followed by third retention force measurement. The final retention force measurement was performed after another simulation of non-axial wearing (15,000 cycles per quadrant).

All samples were subjected to identical artificial aging and investigations. The sum of the wear cycles indicated above was integrated into the test sequence shown in Figure 2.

### 2.6. Microsections

To assess the fit, microsections of a telescopic crown were created from each test series. For this purpose, the corresponding secondary crown was removed from the prosthesis structure. The recemented primary telescope was adapted into the secondary crown with a force of 50 N for a precise fit. This was followed by a complete sheathing of the double crowns with transparent polymer (PMMA, PalaXpress, Kulzer GmbH, Hanau, Germany). A diamond cutting disc (Diamant Instrumente HORICO H 327, Hopf, Ringleb & Co. GmbH & CIE, Berlin, Germany) was used to cut the telescope crowns centrally in the vestibular-oral direction. The cut surfaces were machined using a grinding and polishing machine (Meta Serv 250, Grinder Polisher, Buehler, IL, USA) with silicon carbide abrasive paper, from coarse to fine (CarbiMet Grit 320, 600, 1000, Buehler, IL, USA). Finally, it was smoothed with a polycrystalline diamond suspension (MetaDi SupremePolycrisatline Diamond Suspension, 15 µm, Buehler, IL, USA) on a synthetic rayon cloth (MicroCloth, PSA, 10/PK, Buehler, IL, USA). The microsections were optically analyzed with a light microscope (VMZM-40; 4H-JENA Engineering GmbH, Jena, Germany). The cut was examined for occlusal, labial and lingual discrepancies and for the fit at the cervical primary telescope’s area. Fifty measurements were performed for each measurement area with exception of the cervical measurement area (20 measurements per side).

### 2.7. Statistical Analysis

The statistical analysis was carried out with the SPSS program (version 25.0, IBM, New York, NY, USA). The Kolmogorov-Smirnov test was used to check for normal distribution. The comparison of the individual test series against each other was carried out with the Mann-Whitney U test. Within the series, the differences were verified using the Friedman and Wilcoxon test.

## 3. Results

**Figure 3:**
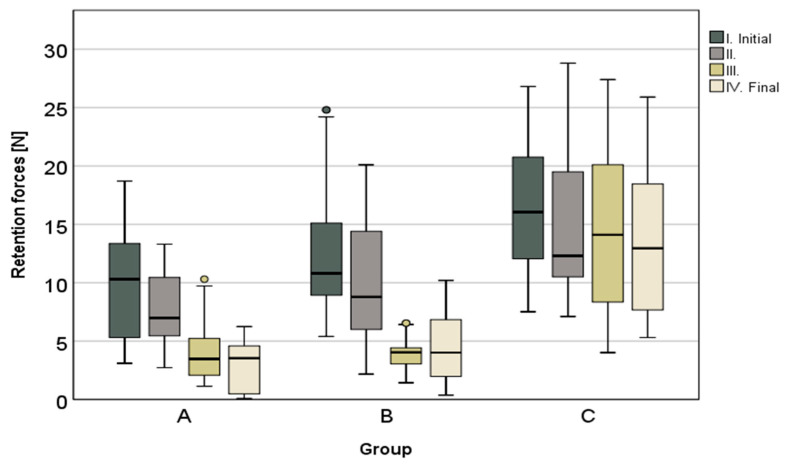
Box plots; median retention force values of all groups at each time of measurement.

The results of the pull-off tests are shown in detail in Table 2 and Figure 3. The telescopic crowns fabricated by CAD-CAM showed, on average, the highest retention forces in the initial and final stages and the lowest retention force loss (*p* < 0.001), with 16.4% over the entire investigation period (artificial aging included). The lowest median values for all measurements were found within group A—the conventional high—noble metal telescopic crowns. In this test series (group A), the retention force decreased from initial to final by 69.7%. A similarly high retention force loss of 63.5% was observed in the non-precious metal conventional telescopic crowns. The initial and final force was increased by ~2–3 N. In both cases, a continuous loss of retention force was observed, which was verified as significant in all intermediate steps (Table 2). With regard to the CAD-CAM fabricated series, only between the measuring points after 15,000 insertion and removal cycles and the final stage could a significant drop of the retention forces be observed. Additionally, there was a significant reduction in the retention forces after multiple wear situations (see Table 2).

With the exception of the measuring point after 15,000 insertion and removal cycles (*p* = 0.472) between groups A and B, significant differences (*p* < 0.001) were found in the comparisons of the test series against each other (groups A, B and C) at each measuring point (I.–IV.).

**Figure 4:**
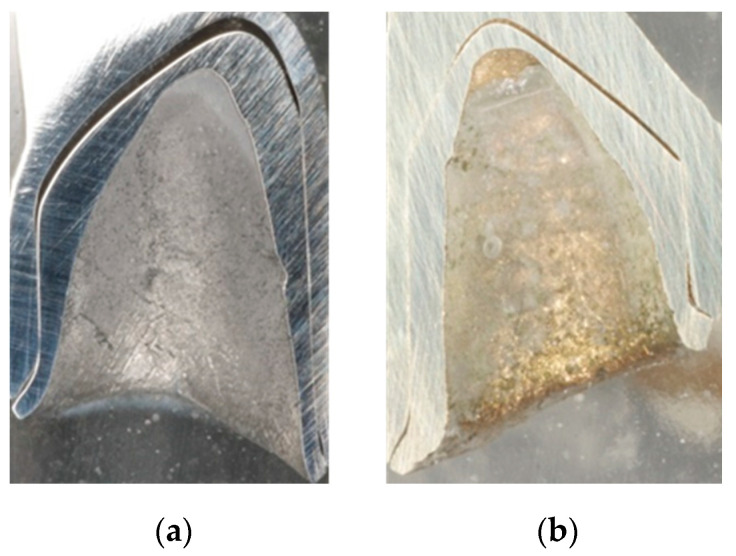
Overview images of microsections. (**a**) milled non-precious metal telescope; (**b**) cast Au telescope.

**Figure 5:**
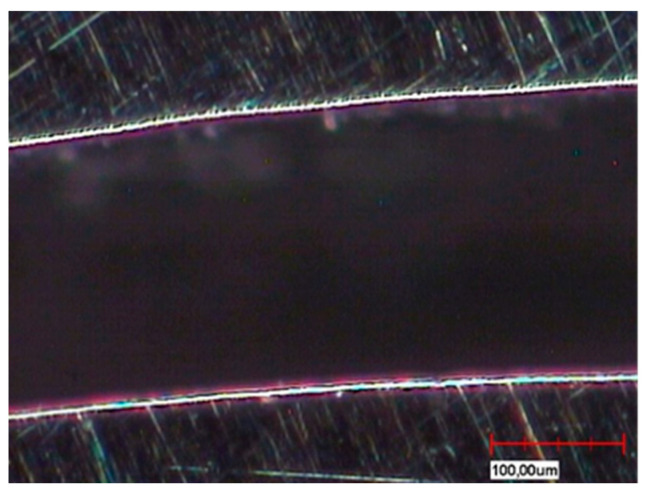
Cut surface of milled non-precious metal telescope, occlusal gap ~220 µm.

**Figure 6:**
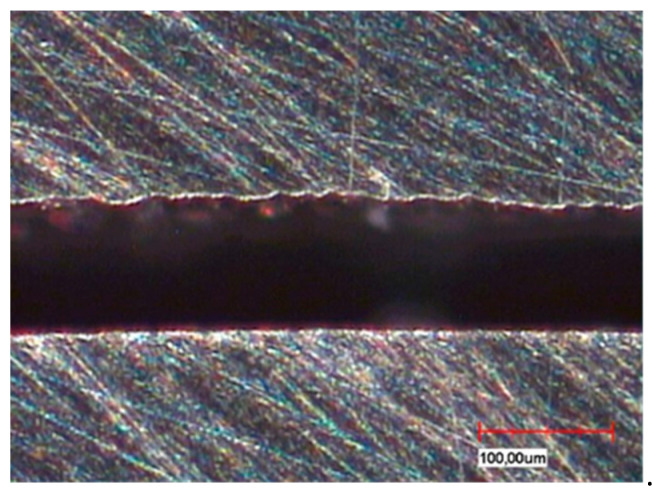
Cut surface of cast Au telescope, occlusal gap ~110 µm and comparatively wavy shape of the inner surface of the secondary crown.

**Figure 7:**
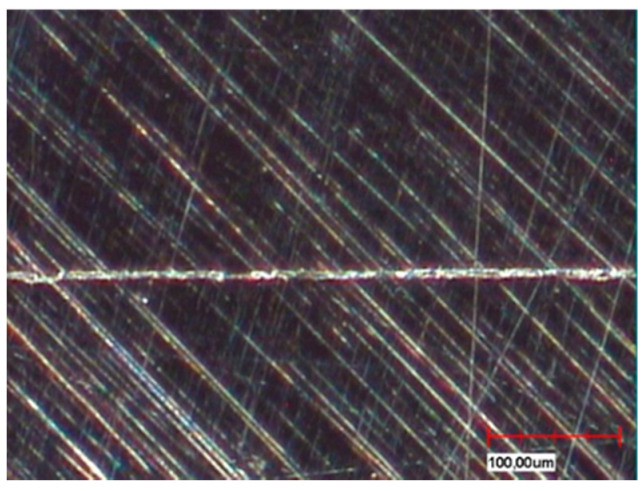
Cut surface of milled non-precious metal telescope (lingual, complete contact surface at parallel milling).

**Figure 8:**
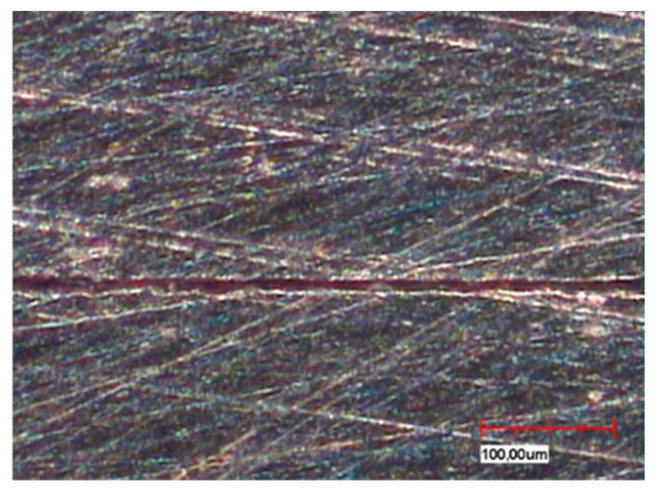
Cut surface of Au telescope (vestibular, contact surface interruption ~10 µm).

The different fabrication methods are also partly reflected in the microsections in the sample cross-section (Figure 4a,b). Depending on the in-house selected design parameters, an average gap of 220 µm is a clearly visible occlusal gap between the primary and secondary crowns of the CAD-CAM-manufactured double crowns (Figure 5). This gap is significantly smaller (almost 100 µm) and visible in the area of the cervical step (Figure 6). In contrast to this, the parallel milled surfaces touch each other completely (Figure 7). The telescope crowns of groups A and B show a similar microsection. However, the occlusal gap is significantly narrower and the inner surfaces of the secondary telescope tend to have more irregular surfaces (Figure 6). In the area of the parallel milling, the contact surfaces show partial interruptions and narrow gaps in the range of 10 to 20 µm (Figure 8).

## 4. Discussion

The null hypothesis, stating that there were no significant differences in the retention forces between identically designed telescopic crowns fabricated by different methods, had to be rejected. The conventionally cast telescopic crowns, both in the version with high gold content and in the non-precious metal version, showed significantly higher retention force losses in total as well as within the individual wear simulations than the CAD-CAM-fabricated telescopic crowns. The measurement methods and test apparatus were discussed in detail and have proven their worth in comparison to measurements in the relevant literature [6].

Digital processes offer a more efficient fabrication option, especially for materials that are difficult to process conventionally. In 2008, there was already an investigation by Shimakura et al. on conical crowns made of titanium and fabricated using CAD-CAM with different parameters in terms of design and occlusal gap dimensions [7]. Also, studies on CAD-CAM-fabricated double crowns in combination with the high-performance plastic PEEK or with zirconium dioxide are listed in the literature [10]. However, the latter studies did not show any reference series regarding the conventional material combinations and they mostly refer to experimentally simplified single stumps. Therefore, results cannot be compared with the available literature and cannot be interpreted as a tendency concerning prosthesis pull-off forces.

The surface morphology (Figure 4, Figure 5, Figure 6, Figure 7 and Figure 8) of the corresponding surfaces of the double crowns strongly influences their adhesiveness [11]. In all three groups of the study, the outer surfaces of the primary crowns were high gloss polished [7]. The morphology of the inner surfaces of the secondary crowns is, therefore, particularly relevant for the comparison. Those of groups A and B were cast and manually machined, while those of group C were milled out in a two-stage process. The resulting morphology and the mechanism of the fit can be derived from the microscopic micrographs (Figure 8, explicitly Figure 6).

With conventional fabrication, the inner surfaces must be cleaned from the investment material and the oxide layer after casting. In the study, this was done by blasting the contact surface with an aluminum dioxide powder at 2 or 4 bar. In addition, the secondary crowns must be fitted onto the primary crowns. For this purpose, excessive contact points are removed from the inner surface with rubber polishers or milling machines. The result is an inhomogeneous surface with a joining gap of varying width and correspondingly irregular contact surfaces. The retention or friction of the conventionally fabricated telescopic crowns is ensured by means of recurring elevations and punctual contacts. By approximating the friction parameters in the molecular range, reversible cold welds can also contribute to an increase in retention. Uniform joint gaps or surfaces are difficult or impossible to achieve with conventional casting technology [7]. Apart from the friction and the joint pressure that prevails depending on the fit tolerance, alloy-specific properties, in particular the moduli of elasticity, also play an important role in groups A and B. The outer surfaces of the primary crowns and the inner surfaces of the secondary crowns are subject to elastically reversible deformations as well as plastically irreversible deformations during chewing movements and during incorporation and removal. With a lower modulus of elasticity, the force required for plastically irreversible deformations is lower. In group B, the modulus of elasticity (200 GPa) is twice as high as in group A (100 GPa) and can therefore be the cause of the slightly higher retention forces.

In principle, depending on the fit, the plastically deformed surfaces can lead to a short-term increase in the contact surfaces and thus contribute to an increase in the retention force; in the case of a weaker fit, it contributes to a reduction of the contact surfaces and thus generates a loss of retention force. This mechanism is mainly active initially in wear simulations or clinically after a short wearing time of a double crown prosthesis due to an increase in retention forces.

In the case of CAD fabrication, fit was adjusted in this study by remilling in the 5 µm range over the entire joint gap and not selectively. The result is a uniform, less rough surface with a consistently defined joint gap (Figure 5 and Figure 7). To create an optimum surface morphology using CAD (Figure 5 and Figure 7), besides precision of the milling process, the selected fit parameters and ideal tools, milling path strategy is decisive [10]. In this study, optimized strategies of the milling center were used over years, so a generalization to all CAD processes would not be correct. However, it shows that a comparatively constant retention force can be achieved by optimally set parameters. Plastic deformations in the form of micro welds probably had less influence on the retention behavior in group C—especially because the alloy used with its high modulus of elasticity (250 GPa) is contrary to this theory. The adhesion behavior tends to correlate more with that of galvanic double crowns [12]. Supported by the comparatively large occlusal gap (see Figure 5), it is possible that a negative pressure will occur in the occlusal gap when loosening the secondary crown. The compensation takes place via a delayed salivary flow in the area of the parallel surfaces between the primary and secondary crown. Resulting flow resistances (Hagen-Poiseuille law) between the contact surfaces increase the adhesion. A very thin capillary-like gap is necessary for this principle. The adhesion is increased by adhesion forces that are created by the respective salivary films within the joining surfaces. In this case adhesion increases the smoother the corresponding surfaces are. 

In addition, the CAD selected fit geometry with the increased gap dimensions in the occlusal area and at the stage of the primary telescope (Figure 4a) offers a kind of retention reservoir. Only in accordance with the conical crown principle, in which the expansion of the secondary crown is essential, the occlusal gap presumably leads to an increase in surface resistance also among the 0° milled telescopes. Shimakura et al. demonstrated on CAD-CAM-fabricated conical crowns made of titanium that the retention forces also increase with an increasing occlusal gap [7]. In the case of the double crowns fabricated by CAD-CAM in this study, the surface resistance is increased less by the wedge effect than by the wear over the wearing period, accompanied by a stronger joint of both crowns, whereas a sufficient retention force is ensured over the tested period. It can also be assumed that even with primary telescopes milled at 0°, a slight deformation of the secondary telescope occurs due to correspondingly high masticatory forces [13,14,15]. 

Due to the small number of samples, the significance of these results must be considered limited. Further studies focusing on the fit between primary and secondary crowns in relation to changed CAD-CAM parameters and retention behavior are necessary to verify the results obtained in the present study.

There are several clinical studies available investigating the long–term behavior of double crown–retained removable partial dentures [16,17,18]. However, due to varying study parameters and target criteria these studies are only partially comparable. Thus, comparable to our previous investigation, the aim of this study was to simulate a distinct wear situation under conditions as close to reality as possible [6]. According to the literature, all tests were performed in a salivary bath [19,20,21]. Based on the assumption that removable partial dentures are removed and inserted three times a day on average, 15,000 cycles of axial loading were performed representing a wearing time of 14 years [6]. In-vivo, extra-axial loading is assumed to accelerate wear [19]. Thus, extra-axial loading was performed representing chewing movements. In the literature, most studies simulated a period of 5 years’ use by performing 1.2 million loading cycles [19,21]. Due to economic reasons, the number of cycles had to be reduced to 50,000 chewing cycles in this study [6]. At this point it should be considered that no investigation was found that systematically evaluated the influence of the number of extra-axial loading cycles on the retention force of removable partial dentures.

In order to reduce systematic bias, the fabrication of double crowns was performed directly on the stumps and the fixed primary crowns. Thus, fabrication-related failures could be minimized, impression-related mistakes excluded and reattachments avoided [6]. To avoid undesirable transverse forces, an ideal axial insertion direction was chosen. The removal of the prosthesis was performed using three trigonally arranged steel wires (26 mm), to avoid torsion and transverse forces during deduction. Previous in vitro studies have attempted to minimize this risk by using long steel wires (0.5 m) [22,23]. The removal velocity of 50 mm/min used was in accordance with previous studies reporting this to be the regular clinical removal velocity [24]. The load of 50 N used was postulated to be optimal by Ohkawa et al. and Chung et al. [25,26].

However, complex natural jaw and chewing movements as well as loading during mastication cannot be simulated in-vitro. Thus, in vitro studies can only provide an approximation of reality and their results must be critically assessed in terms of their meaningfulness. Another restriction of the present investigation is that the number of samples was limited by the production costs. Thus, the significance of the measured values is additionally limited from a statistical point of view.

## 5. Conclusions

The results of the study confirm that the retention forces of double crown prostheses depend on the selected material combination, as well as the fabrication process. In an optimized CAD-CAM process, the milled non-precious metal double crowns showed the highest retention forces with a comparatively continuous retention force behavior during the wear simulation.

This may be attributed to a better fit with a consequently narrower joint gap and a comparatively wider occlusal gap between the primary and secondary crowns. Conventionally cast, identical double crowns with a comparable composition of the alloy used showed lower retention forces with a significant loss of retention force.

## Figures and Tables

**Table 1:** Telescopic crowns and components used according to the manufacturer´s specifications.

Group	Telescopic Crowns 0°	Material/Composition	
	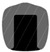	Coping	Secondary Crown
**A**	Conventional fabricated *high-noble metal*	Bio RD1 (Degunorm) ^a^ *Au 73.8%; Ag 9.2%; Pt 9.0%; Cu 4.4%; Ir 0.1%; Zn 2.0%; In 1.5%*	Bio RD 1 (Degunorm) ^a^ *Au 73.8%; Ag 9.2%; Pt 9.0%; Cu 4.4%; Ir 0.1%; Zn 2.0%; In 1.5%*
		Elastic modulus = 100 GPa	
**B**	Conventional fabricated *non-precious metal*	Okta-C ^a^ *Co 61.1 wt%; Cr 32.0 wt%; Mo 5.5 wt%; Si 0.7 wt%; Mn 0.7 wt%*	Okta-M VS ^a^ *Co 61.6 wt%; Cr 30.0 wt%; Mo 6.5 wt%; Si 0.8 wt%; Mn 0.8 wt%; C 0.3 wt%*
		Elastic modulus = 200 GPa	
**C**	CAD-CAM fabricated *non-precious metal*	Organic CoCr ^b^ *Co 63%, Cr 29%, Mo 6%, Si, Mn, Nb, Fe < 1%*	Organic CoCr ^b^ *Co 61.5%, Cr 27.75%, W 8.45%, Si 1.61%, Mn 0.25%, Fe 0.2%, Others < 0.1%*
		Elastic modulus = 250 GPa	Elastic modulus = 240 GPa

^a^ SAE DENTAL VERTRIEBS GMBH, Bremerhaven, Germany. ^b^ Organical CAD/CAM GmbH, Berlin, Germany.

**Table 2:** Mean, standard deviation (SD), minimum (min), maximum (max) and percentile with median retention force values in groups A to C.

		Group A Conventional High-Noble Metal				Group B Conventional Non-Precious Metal				Group C CAD-CAM Non-Precious Metal			
Time of measurement		I.	II.	III.	IV.	I.	II.	III.	IV.	I.	II.	III.	IV.
**Mean**		9.6	7.5	4.2	2.9	12.5	10.2	3.8	4.6	16.2	15.1	14.4	13.6
**SD**		4.4	2.7	2.5	2.0	5.1	5.2	1.1	2.9	5.7	5.3	6.9	6.2
***p*** **-value**		*p* < 0.001				*p* < 0.001				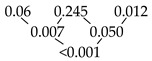			
within the groups		in all comparisons				in all comparisons							
**Min**		3.1	2.7	1.1	0.1	5.4	2.2	1.4	0.4	7.5	7.1	4.0	5.3
**Max**		18.7	13.3	10.3	6.2	24.8	20.1	6.54	10.2	26.8	28.8	27.4	25.9
**Percentile**													
	25	5.3	5.4	2.1	0.5	8.9	6.0	3.0	1.9	12.0	10.5	8.3	7.3
	50 median	10.3	7.0	3.5	3.5	10.8	8.8	4.0	4.0	16.1	12.2	14.1	13.0
	75	13.4	10.5	5.3	4.6	15.2	14.6	4.4	7.0	21.0	19.6	21.1	18.5
